# Vaccination

**Published:** 1913-11

**Authors:** 


VACCINATION. Wm. Hanna, M. A., M. D., D. P. H., Asst. Medical Officer of Liverpool, in a recent book entitled Studies in Small Pox and Vaccination, reports 2280 cases of small pox. The aggregate mortality was 161, about 7½ per cent. The mortality of 220 that had never been vaccinated ranged from 25 to 40 per cent. of 943 which had been vaccinated only in infancy, it was 3 per cent. No cases occurred in vaccinated children under three years of age, and no deaths in vaccinated persons under 20.

